# Accurate and fast estimation of taxonomic profiles from metagenomic shotgun sequences

**DOI:** 10.1186/gb-2011-12-s1-p11

**Published:** 2011-09-19

**Authors:** Bo Liu, Theodore Gibbons, Mohammad Ghodsi, Todd Treangen, Mihai Pop

**Affiliations:** 1 Center for Bioinformatics and Computational Biology, University of Maryland, College Park, MD 20742, USA; 2 Department of Computer Science, University of Maryland, College Park, MD 20742, USA; 3 Biological Sciences Graduate Program, University of Maryland, College Park, MD 20742, USA

## Background

A major goal of metagenomics is to characterize the taxonomic composition of an environment. The most popular approach relies on 16S rRNA sequencing; however, this approach can generate biased estimates owing to differences in the copy number of the gene, even between closely related organisms, and owing to PCR artifacts. In addition, the taxonomic composition can also be determined from metagenomic shotgun sequences by matching reads against a database of reference sequences. One major limitation of the computational methods that have been used for this purpose is the use of a universal classification threshold for all genes at all taxonomic ranks.

## Methods

We present a novel taxonomic profiler for metagenomic sequences, MetaPhyler [1], which relies on 31 phylogenetic marker genes as a taxonomic reference. Because genes can evolve at different rates and because shotgun reads contain gene fragments of different lengths, we propose that better classification results can be obtained by tuning the taxonomic classifier to the length of the gene fragment, to a particular gene and to the taxonomic rank. Our classifier uses different thresholds for each of these parameters, and these thresholds are automatically learned from the taxonomic structure of the reference database.

## Results

We have randomly simulated about 300,000 DNA sequences of 60 bp and about 70,000 DNA sequences of 300 bp from phylogenetic marker genes. Table 1 shows the performance of the phylogenetic classifications from MetaPhyler, PhymmBL [2], MEGAN [3] and WebCARMA [4]. The query sequence itself was removed from the reference dataset when running the programs. The sensitivity of MetaPhyler is significantly higher than that of the other tools in all situations because our classifier is explicitly trained at each taxonomic rank.

**Table 1:** Comparison of sensitivity and precision.

Sequence length	Parameter	Taxonomic rank	MetaPhyler (%)	PhymmBL (%)	MEGAN (%)	WebCARMA (%)
60 bp	Sensitivity	Genus	33.45	18.18	15.49	22.66
		Family	54.22	38.75	24.52	25.10
		Order	59.59	49.36	31.74	28.22
		Class	70.72	62.86	50.78	32.12
		Phylum	75.30	68.88	64.19	34.65
	Precision	Genus	96.38	94.42	90.72	35.22
		Family	97.45	97.66	97.18	45.71
		Order	97.39	97.65	98.10	52.51
		Class	98.27	98.15	99.11	66.15
		Phylum	98.83	99.06	99.56	72.90
300 bp	Sensitivity	Genus	52.39	42.97	20.89	45.96
		Family	70.17	58.81	34.27	52.49
		Order	78.09	66.72	45.24	58.56
		Class	84.52	75.42	61.06	62.70
		Phylum	91.18	76.78	81.36	66.49
	Precision	Genus	97.90	96.16	96.09	77.63
		Family	99.14	99.07	99.19	88.69
		Order	99.15	99.15	99.21	92.67
		Class	99.34	99.34	99.57	95.43
		Phylum	99.64	99.64	99.80	96.58

In addition, we have created a simulated metagenomic sample comprising five genomes. Table 2 shows the taxonomic profiles estimated by different approaches. In this setting, MetaPhyler also outperforms the other approaches by more accurately reconstructing the true taxonomic distribution.

**Table 2:** Comparison of taxonomic profile estimations.

Genus	True (%)	MetaPhyler (%)	PhymmBL (%)	MEGAN (%)	WebCARMA (%)
*Bifidobacterium*	50.0	50.0	34.3	32.8	34.3
*Bacteroides*	20.0	20.4	32.1	34.3	33.8
*Staphylococcus*	10.0	10.2	9.4	9.1	8.9
*Enterococcus*	10.0	10.1	9.0	7.3	10.4
*Clostridium*	10.0	9.4	11.8	12.1	12.6
Other	0.0	0.0	3.6	4.4	0.1

## Conclusions

We have introduced a novel taxonomic classification method for analyzing the microbial diversity from whole metagenome shotgun sequences. Compared with previous approaches, MetaPhyler is more accurate at estimating the taxonomic profile, especially when taking into account the actual abundance of individual taxonomic groups.
